# Heart Rate Responses to Autonomic Challenges in Obstructive Sleep Apnea

**DOI:** 10.1371/journal.pone.0076631

**Published:** 2013-10-23

**Authors:** Paul M. Macey, Rajesh Kumar, Mary A. Woo, Frisca L. Yan-Go, Ronald M. Harper

**Affiliations:** 1 University of California at Los Angeles School of Nursing, University of California Los Angeles, Los Angeles, California, United States of America; 2 Brain Research Institute, David Geffen School of Medicine at University of California Los Angeles, University of California Los Angeles, Los Angeles, California, United States of America; 3 Department of Neurobiology, David Geffen School of Medicine at University of California Los Angeles, University of California Los Angeles, Los Angeles, California, United States of America; 4 Department of Neurology, David Geffen School of Medicine at University of California Los Angeles, University of California Los Angeles, Los Angeles, California, United States of America; Tokai University, Japan

## Abstract

Obstructive sleep apnea (OSA) is accompanied by structural alterations and dysfunction in central autonomic regulatory regions, which may impair dynamic and static cardiovascular regulation, and contribute to other syndrome pathologies. Characterizing cardiovascular responses to autonomic challenges may provide insights into central nervous system impairments, including contributions by sex, since structural alterations are enhanced in OSA females over males. The objective was to assess heart rate responses in OSA versus healthy control subjects to autonomic challenges, and, separately, characterize female and male patterns. We studied 94 subjects, including 37 newly-diagnosed, untreated OSA patients (6 female, age mean±std: 52.1±8.1 years; 31 male aged 54.3±8.4 years), and 57 healthy control subjects (20 female, 50.5±8.1 years; 37 male, 45.6±9.2 years). We measured instantaneous heart rate with pulse oximetry during cold pressor, hand grip, and Valsalva maneuver challenges. All challenges elicited significant heart rate differences between OSA and control groups during and after challenges (repeated measures ANOVA, *p*<0.05). In post-hoc analyses, OSA females showed greater impairments than OSA males, which included: for cold pressor, lower initial increase (OSA vs. control: 9.5 vs. 7.3 bpm in females, 7.6 vs. 3.7 bpm in males), OSA delay to initial peak (2.5 s females/0.9 s males), slower mid-challenge rate-of-increase (OSA vs. control: −0.11 vs. 0.09 bpm/s in females, 0.03 vs. 0.06 bpm/s in males); for hand grip, lower initial peak (OSA vs. control: 2.6 vs. 4.6 bpm in females, 5.3 vs. 6.0 bpm in males); for Valsalva maneuver, lower Valsalva ratio (OSA vs. control: 1.14 vs. 1.30 in females, 1.29 vs. 1.34 in males), and OSA delay during phase II (0.68 s females/1.31 s males). Heart rate responses showed lower amplitude, delayed onset, and slower rate changes in OSA patients over healthy controls, and impairments may be more pronounced in females. The dysfunctions may reflect central injury in the syndrome, and suggest autonomic deficiencies that may contribute to further tissue and functional pathologies.

## Introduction

Obstructive sleep apnea (OSA) affects over 20 million adults in the United States alone, and is associated with multiple serious health consequences, including deficits related to central nervous system (CNS) function, such as elevated sympathetic tone, high levels of depressive and anxiety symptoms, daytime sleepiness, and cognitive difficulties [1]–[3]. The CNS symptoms are accompanied by changes to brain structure and function, with both respiratory and central autonomic regulation being affected [4]–[11]. One difficulty with treating OSA is that most CNS-related symptoms improve only partially, or not at all with the gold standard intervention, continuous positive airway pressure (CPAP). Autonomic functioning, in particular, is of concern as hypertension, a primary comorbidity of OSA, is not relieved by CPAP [12]–[15], suggesting that deleterious processes that underlie the high blood pressure in OSA continue to operate independently from the breathing pauses (apneas and hypopneas). Those deleterious processes may have developed from changes in autonomic regulatory brain areas during the course of OSA development, leading to impairments in both sustained and dynamic autonomic regulation.

If normal cardiovascular responses to typical autonomic challenges (straining, vestibular alterations from changes in body position, temperature change) are impaired, then tissue is at risk of being starved from inadequate blood flow. Brain tissue, in particular, is sensitive to acute periods of ischemia and rapid reperfusion [16], [17]. Thus, a negative feedback cycle of brain structural changes leading to impaired autonomic regulation, which then contributes to inadequate cardiovascular responses to daily demands, may result in further brain tissue changes.

Exaggerated and unvarying sympathetic tone in OSA suggests some autonomic circuits are disrupted, but the particular systems affected are unclear [18], [19]. Dynamic cardiovascular responses may give some indication of which systems are impacted. Cardiovascular responses arise from different autonomic pathways, depending upon the stimuli. A voluntary limb muscle contraction elicits heart rate increases through integration of autonomic regulatory networks with sensory and motor components of the cortex, cerebellum, and basal ganglia [20]–[22]. Temperature change (cold) induces autonomic responses through medullary, hypothalamic and insular cortex areas [15], [20]. A pain stimulus triggers sympathetic action through integration of sensory input within medullary, mid-brain, thalamic, and insular cortex regions [23], [24]. Changes in thoracic and upper airway pressure result in signaling that elicits both sympathetic and parasympathetic activation with associated heart rate changes [20]. Using a variety of autonomic challenges therefore provides an opportunity to evaluate integrity of separate pathways used in mediating those challenges.

An important confound in autonomic assessment in OSA is that women with OSA are more affected by the condition than men. The disorder is less prevalent in females than males, with women showing an incidence of the syndrome half that found in men [25]. However, brain structures are more impacted in female than male patients [26]. The disorder differs between the sexes in other ways, including both physical and psychological symptoms [27]–[33]. Disease severity, as measured by apneic events per hour (apnea/hypopnea index, AHI), tends to be lower in females, but women are symptomatic at lower AHI levels [27]. A generalization is that women with OSA present with a greater variety of symptoms, including depression and anxiety [28], [29], [34], [35]. Thus, the overall impression is that the phenomenon of obstructive breathing pauses does not produce the same outcomes in men as in women. Taken with the known sex differences in brain structure and autonomic function in the general population, any assessment of autonomic function will benefit from separation by sex.

Our objective was to describe heart rate responses to three autonomic challenges in OSA and control populations, with additional separation by sex. We selected the cold pressor, hand grip, and Valsalva maneuver challenges, since these challenges elicit a pressor response with sympathetic activation, but via differing mechanisms. The cold pressor challenge, having a foot placed in water close to freezing, involves temperature and pain signaling, with no voluntary component. The hand grip task, a voluntary forceful squeeze by the hand, involves voluntary motor action and muscle contraction. The Valsalva maneuver, a forced expiratory effort creating a sustained thoracic pressure, involves a combination of voluntary motor action, pressure signaling from chest and upper airway receptors, and activation of vascular pressure sensors. The maneuver elicits a sequence of both sympathetic and parasympathetic actions. This combination of tasks allows focus on unique deficits that might arise in the autonomic nervous system. One aim was to assess the similarities and differences in heart rate responses in OSA patients with respect to healthy control subjects, with additional consideration by sex. We also aimed to test the hypothesis that heart rate patterns to these challenges in OSA patients would be muted, consistent with structural alterations to brain rostral regulatory areas, and delayed, expected with cerebellar injury earlier-described [5], [6], [11]. We further hypothesized that impaired timing of OSA response patterns, relative to controls, would be parallel in males and females, but that female responses would show greater magnitude of separation from controls than males, given the additional structural brain alterations in that group [26].

## Methods

### Ethics Statement

All procedures were in accordance with the Declaration of Helsinki and approved by the UCLA Institutional Review Board, and subjects provided written, informed consent.

### Subjects

We recruited OSA patients from the University of California at Los Angeles (UCLA) Sleep Laboratory, and control subjects from the Los Angeles community. No OSA subjects were treated for the sleep disorder, and all were recently-diagnosed, as determined by full overnight polysomnography. The OSA patients were classified as moderate or severe, based on an AHI of 15 events/hour or higher [36]. Control participants reported good health, and no sleep disorders. All control subjects were administered a screening survey for the presence of symptoms possibly from OSA (sleepiness, snoring, report from bed partner about snoring or obstructive events), and from this screening, five subjects were administered a full sleep study, resulting in one being diagnosed with moderate OSA, two being diagnosed with mild OSA (AHI≥5 and AHI<15), and two being confirmed as having no sleep disorder. No subjects had a history of psychiatric disorders, cardiovascular disease, stroke, or other major illness. Control participants were recruited via fliers and word-of-mouth on the university medical center and campus. No OSA or control participants were using medications that could affect autonomic function, such as psychotropic or cardiovascular agents. Scanner limitations precluded patients with metallic implants or weight over 125 kg. Sex was categorized based on subject responses to a questionnaire (“Check female or male”), and age was calculated based on reported birthdate. These subjects overlap samples from other studies [6], [26], and the data presented in this paper were collected within a larger project of neuroimaging assessment of autonomic function. The heart rate findings are complex and extensive; thus, the imaging data will be presented separately.

### Psychological Symptoms

Psychological symptoms were measured, as these are associated with autonomic alterations, especially anxiety and depression. We administered the Beck Depression Inventory-II (BDI) for depressive symptoms [37], [38] and the Beck Anxiety Inventory (BAI) for anxiety symptoms [39]. Daytime sleepiness was measured with the Epworth Sleepiness Scale (ESS) [40], and sleep quality with the Pittsburg Sleep Quality Index (PSQI) [41].

### Protocol

Since these data are a part of a large neuroimaging study, the challenges and heart rate recordings were performed in an MRI scanner. Subjects were asked to refrain from coffee and other substances with stimulants for the 24 hours prior to the study. After completion of screening and enrolment, subjects practiced the Valsalva maneuver and the hand grip challenges. For the Valsalva maneuver, subjects were instructed to wait for a visual signal (blue light), then take a deep breath and expire from their chest until they achieved a pressure of 30 mmHg (indicated by a green light). They maintained this pressure until the end of the 18 s challenge period (indicated by blue light being turned off), and they would then breathe normally. Once subjects could perform the task, they were moved into the scanner, where they repeated the challenge. For the hand grip challenge, subjects were instructed to squeeze an inflated bag with the right hand. They were initially directed to briefly squeeze at maximum as a reference. The challenge consisted of a 16 s grip period (indicated to the subject by a blue light) at a subjective 80% of maximum. Subjects practiced first outside, then inside the scanner.

After practice, subjects were scanned in the supine position with anatomical protocols, which provided time to allow their physiologic functions to return to a baseline state. Following a minimum of 30 minutes of structural scanning, the autonomic testing protocols were started, consisting of a 436 s period that included a baseline (100 s) followed by four Valsalva challenges 60 s apart, and a final 60 s recovery. A similar pattern was followed for the hand grip challenge. The cold pressor challenge consisted of a 100 s baseline, 60 s right foot immersion to approximately ankle height in cold water (4°C, verified by digital thermometer), and a 60 s recovery. Two investigators held the foot at all times, and lifted the foot into and out of the water at the appropriate times.

### Measurements: Physiologic Signals

Physiologic signals were recorded on a laptop with a digitizer synchronized with the scanning signal. A pulse oximeter (Nonin 8600) with a sensor on the left index finger was used to measure the plethysmographic waveform. The expiratory pressure for the Valsalva maneuver was measured with a pressure sensor outside the scanner room, connected via a tube into which a subject created expiratory pressure. Change in hand grip pressure was measured with a pressure sensor connected to the compressible bag which the subjects’ squeezed; this signal was a relative measure, and categorized into the presence or absence of pressure.

### Data Checking: Task Performance and Data Quality

Each autonomic task performance was verified by inspecting the physiologic signals. For the Valsalva maneuver, subjects who did not achieve a sustained 30 mmHg pressure for the duration (and no longer) of each of the four challenges were excluded. The signals were checked to ensure all subjects exerted force during the four hand grip periods. Since the cold pressor was a passive challenge, no series were excluded.

### Analysis

The instantaneous heart rate was derived from peaks in the plethysmographic waveform, with motion artifacts excluded. We used repeated measures ANOVA (RMANOVA) to assess heart rate time trends in a mixed linear model implemented in SAS software (“proc mixed”) [42]. Random effects across subjects were accounted for, and time was binned into baseline (single bin) and 1 s epochs. Overall model effects were tested, and according to the Tukey-Fisher criterion for multiple comparisons, only significant models and effects were assessed for within-group (i.e., increases or decreases relative to baseline) or between-group (i.e., differences between OSA and control in magnitude of response) effects at each time-point in the challenge and recovery. The analyses of the repeated tasks (Valsalva and hand grip) were performed on data grouped over the four challenges. Given the potentially detailed nature of patterns of time trend differences, we also calculated indices representing simplified representations of the significance within and between group effects. Some, such as the Valsalva ratio and tachycardia ratio, are standard representations; whereas, others were empirically derived. The indices were distinguished as representing differences in magnitude (heart rate increase from baseline to peak, sustained heart rate increases measured by area-under-the-curve), and timing (delayed peak, or different slope during periods of heart rate increase). The latter (delay) was based only on the mean signal, as the individual time-scale was poor (due to the timing resolution of heart rate being a function of the each beat, in the order of magnitude of 1 measure/second). Analyses of demographic and neuropsychologic variables, which may not be normally distributed, were performed with both parametric and the non-parametric Kruskal-Wallis one-way ANOVA procedures.

## Results

### Subjects

Subject characteristics are shown in Table 1. Of 110 subjects originally studied, 94 met the inclusion criteria for data quality and successful performance of the tasks. Reasons for exclusion were: artifact or dropout of the pulse oximetry recording; failure to achieve the target pressure for the Valsalva; and for one subject, excessive duration of strain period for the Valsalva. The numbers per group were: 6 OSA females, 20 control females, 31 OSA males, and 37 control males. Of the included subjects, significant group differences appeared in all symptoms (Table 1), with OSA females showing the highest levels of each symptom (BAI, BDI, ESS, and PSQI). Male subjects showed a trend toward higher severity of OSA than females, with a significant difference appearing in oxygen saturation nadir.

**Table 1:** Subject information across subgroups, with means and standard deviations (±std). Group effects were evaluated with parametric and Kruskal-Wallis ANOVA tests (four-group for age, BMI and psychological measures, and two-group for OSA parameters).

	Female		Male			ANOVA	Kruskal-Wallis
	Control	OSA	Control		OSA	*p* (F-test)	*P (Chi-square)*
N	20	6	37		31		
Age (years)	50.5±8.1	52.1±8.1	45.6±9.2		45.3±8.4	0.06	0.06
BMI (m^2^/kg)	24.1±5.3	32.4±3.2	25.1±2.8		30.2±4.8	<0.001	<0.001
BAI (anxiety)	5.6±6.7	19.8±19.0	3.5±4.3		7.3±7.4	<0.001	0.006
BDI (depression)	5.3±4.7	13.0±11.1	3.7±5.6		8.0±3.8	0.003	0.001
Epworth SS	6.8±3.6	10.2±3.7	4.9±3.7		10.0±4.9	<0.001	<0.001
PSQI	4.4±0.7	10.6±1.1	3.9±0.6		7.7±0.8	<0.001	<0.001
OSA Parameters							
AHI (events/hour)		27.7±15.6			37.4±19.6	0.1	0.2
SAO_2_ nadir		86.0±1.5			77.0±9.2	0.03	0.008
SAO_2_ baseline		94.3±1.5			94.8±2.1	0.8	0.2

### Cold Pressor Summary

**Figure 1:**
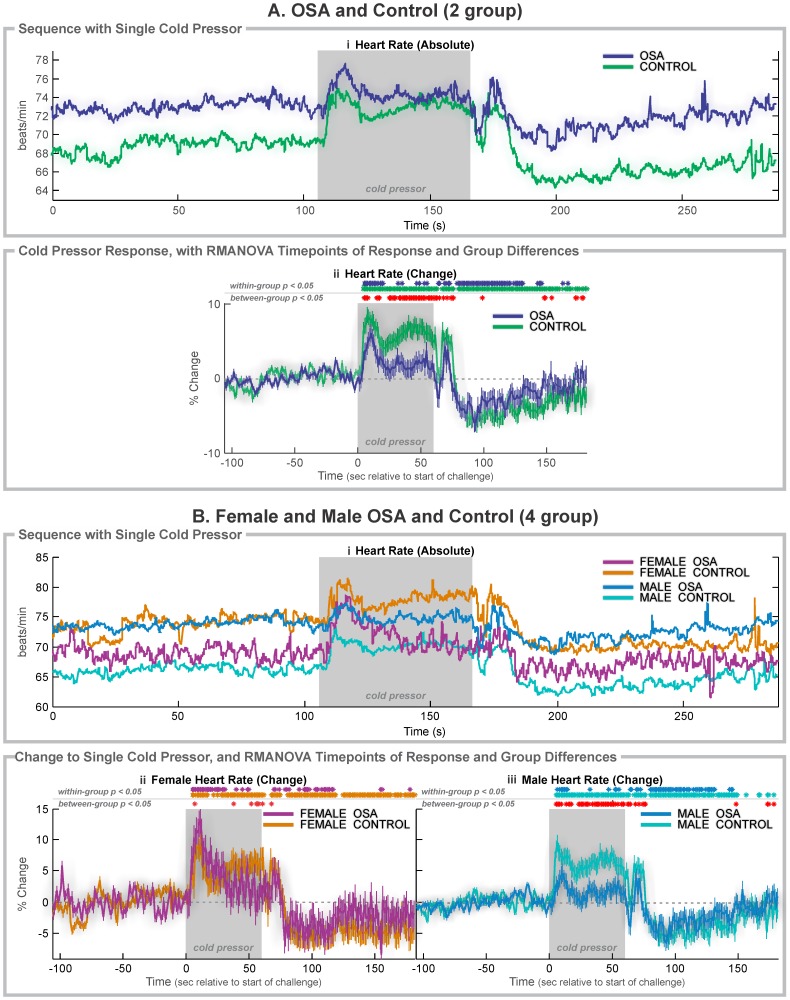
Cold pressor heart rate responses. A: 37 OSA and 57 control subjects; B: 6 OSA and 20 control female subjects, and 31 OSA and 37 control male subjects. Ai, Bi: Raw heart rate during the complete sequence. Aii, Bii, Biii: Change in heart rate relative to baseline (group mean ± SE), with time-points of significant increase or decrease relative to baseline within-group, and time-points of between-group differences (RMANOVA, *p*<0.05). Shaded areas indicate challenge periods.

**Figure 2:**
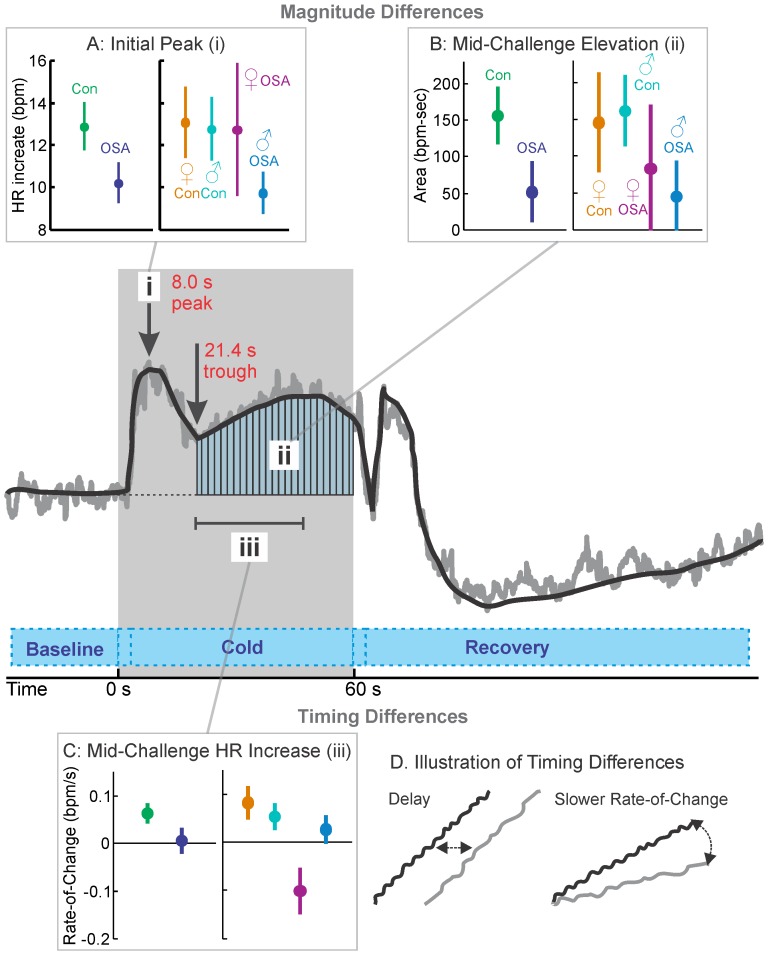
Cold pressor indices. A: heart rate increase from baseline to initial peak (i); B: sustained heart rate elevation during mid-challenge period (area-under curve, ii); C: rate of change (slope) during mid-challenge increase (iii). Time trend graph illustrates the mean heart rate of the 57 subjects in the control group, with a simplified representation (thick black line) overlaid on the measured values (gray line). Shaded area indicates challenge period.

The foot cold pressor challenge elicits both a blood pressure rise through temperature stimulation and, after an initial period of 10–20 seconds, pain sensations in the immersed area. After foot immersion in cold water, both OSA and control subjects showed sustained heart rate increases consistent with sympathetic activation (Fig. 1 & Table 2). The initial 2–4 s of the challenge period was required to move the foot into the water, and a similar time at the end of the challenge was used to remove the foot. These patterns are reflected in the indices (Fig. 2 & Table 3), with lower initial peak values in the OSA, and especially male OSA groups (Fig. 2A), and lower overall elevation (reduced area-under-the-curve in all OSA groups; Fig. 2B). Timing differences appeared with low (or negative for female) OSA rates of increase during the mid-portion of the challenge (Fig. 2C), and delays in the first peak, again with OSA impairments exacerbated in females vs. males (2.5 s vs. 0.9 s).

**Table 2:** Statistics for mixed models implementing RMANOVA.

		Overall Model			Individual Variables					
					Group		Time		Group*Time	
		ChiSquare		*p*	F	*p*	F	*p*	F	*p*
**OSA (N = 37) vs. Control (N = 57)**										
	Cold pressor	3363	<0.0001		1	0.3	22	<0.0001	4	<0.0001
	Hand grip	2066	<0.0001		1	0.3	55	<0.0001	2	<0.0001
	Valsalva	2061	<0.0001		0.1	0.7	305	<0.0001	6	<0.0001
**Female OSA (N = 6) vs. Control (N = 20)**										
	Cold pressor	1091	<0.0001		0.5	0.5	11	<0.0001	1	0.02
	Hand grip	459	<0.0001		0	0.9	10	<0.0001	1	0.009
	Valsalva	1202	<0.0001		3	0.1	68	<0.0001	10	<0.0001
**Male OSA (N = 31) vs. Control (N = 37)**										
	Cold pressor	2480	<0.0001		2	0.1	15	<0.0001	3	<0.0001
	Hand grip	1559	<0.0001		3	0.08	46	<0.0001	2	<0.0001
	Valsalva	1409	<0.0001		0.2	0.7	236	<0.0001	4	<0.0001

**Figure 3:**
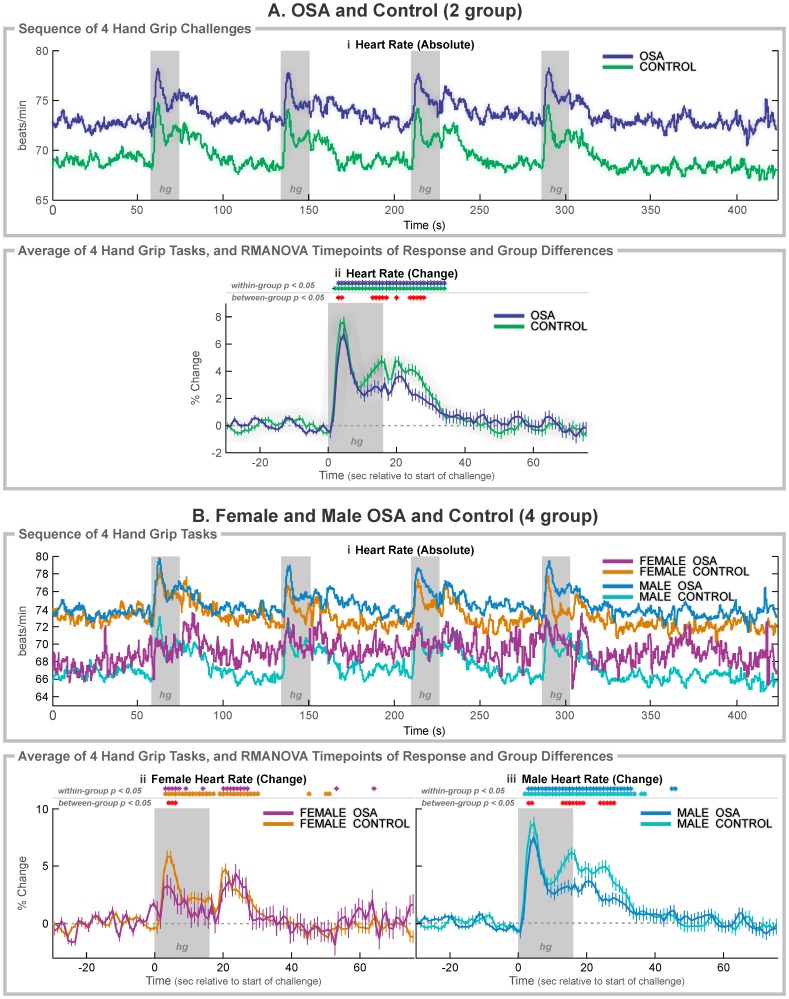
Hand grip heart rate responses. A: 37 OSA and 57 control subjects; B: 6 OSA and 20 control female subjects, and 31 OSA and 37 control male subjects. Aii, Bii: Raw heart rate during the complete sequence. Aii, Bii, Biii: Change in heart rate relative to baseline (group mean ± SE) averaged over 4 challenges, with time-points of significant increase or decrease relative to baseline within-group, and time-points of between-group differences (RMANOVA, *p*<0.05). Gray rectangles (hg) indicate challenge periods.

Models were implemented in the SAS procedure proc mixed [42]. Repeated measures were across time-points, with subject as a random factor. The overall model fit is reported as ChiSquare and p value. For significant overall models (which were all for this data set), variables were tested individually, demonstrating significant time and group-by-time effects in all cases (F statistic and p value reported). Significant within and between group differences at individual time-points are shown in Figs. 1, 3 & 5.

**Table 3:** Indices for each challenge.

Group		Time period	OSA	Control	Female		Male		Figure
					OSA	Control	OSA	Control	
N			37	57	6	20	31	37	
**Cold pressor**									
Initial peak (bpm increase from baseline) ± SEM									Fig. 2 (A)
		0.0–21.4 s	4.7±1.0	5.8±1.1	9.5±3.2	7.3±1.7	3.7±1.0	7.6±1.5	
			ANOVA F = 2.8, *p* = 0.1		ANOVA F = 1.2, *p* = 0.3				
Mid-to-late challenge elevation (area in beats, from bpm*sec)									Fig. 2 (B)
		21.4–60.0 s	45.4±42.4	149.8±40.0	77.8±87.1	148.1±68.7	38.9±48.2	154.5±49.0	
			ANOVA F = 3.1, *p* = 0.08		ANOVA F = 1, *p* = 0.4				
Delay to initial peak (seconds of OSA delay wrt Control, in mean signal)									Fig. 2 (C)
		2.0–21.4 s	2.0 s		2.48 s		0.87 s		
Mid-challenge rate of increase (bpm/sec) ± SEM									Fig. 2 (D)
	21.4–51.4 s		0.0087±0.027	0.0704±0.022	−0.105±0.049	0.085±0.035	0.028±0.030	0.056±0.028	
			ANOVA F = 2.7, *p* = 0.1		ANOVA F = 2.0, *p* = 0.1				
**Hand grip**									
Initial peak (bpm increase from baseline) ± SEM									Fig. 4 (A)
		0.0–8.5 s	4.8±0.7	5.4±0.7	2.6±1.1	4.6±0.9	5.3±0.7	6.0±0.9	
			ANOVA F = 4.5, *p* = 0.03		ANOVA F = 5.2, *p* = 0.002				
Strain elevation (area in beats, from bpm*sec) ± SEM									Fig. 4 (B)
	4.0–16.0 s		33.7±5.9	41.5±5.6	13.7±8.9	27.7±8.7	35.1±6.8	46.3±7.2	
			ANOVA F = 2.9, *p* = 0.09		ANOVA F = 4.1, *p* = 0.007				
Delay to initial peak (seconds of OSA delay wrt Control, in mean signal)									Fig. 4 (C)
1.0–7.0 s			0.19 s		−0.36 s		0.15		
**Valsalva**									
Phase II elevation (area in beats, from bpm*sec) ± SEM									Fig. 6 (A)
5.7–18.0 s			93.4±15.5	124.3±18.1	50.6±15.2	134.6±35.9	103.2±18.0	119.8±20.4	
			ANOVA F = 6.6, *p* = 0.01		ANOVA F = 3.8, *p* = 0.01				
Tachycardia ratio ± SEM									Fig. 6 (TR)
5.7–18 s (challenge)20.0 s baseline			1.17±0.03	1.21±0.03	1.09±0.03	1.21±0.05	1.19±0.03	1.21±0.03	
			ANOVA F = 11.2, *p* = 0.0009		ANOVA F = 5.3, *p* = 0.001				
Valsalva ratio ± SEM									Fig. 6 (VR)
5.7–18 s (challenge)0.0–20.0 s (recovery)			1.26±0.05	1.32±0.05	1.14±0.12	1.30±0.07	1.29±0.05	1.34±0.06	
			ANOVA F = 11.4, *p* = 0.0008		ANOVA F = 6.4, *p* = 0.0003				
Phase II rate of increase ± SEM									Fig. 6 (B)
5.7–18.0 s			1.13±0.16	1.30±0.13	0.62±0.23	1.25±0.20	1.23±0.18	1.34±0.17	
			ANOVA F = 2.0, *p* = 0.2		ANOVA F = 3.4, *p* = 0.02				
Delay to phase III peak (seconds of OSA delay wrt Control, in mean signal)									Fig. 6 (C)
1.4–5.4 s (recovery)			1.08 s		0.68 s		1.31 s		

**Figure 4:**
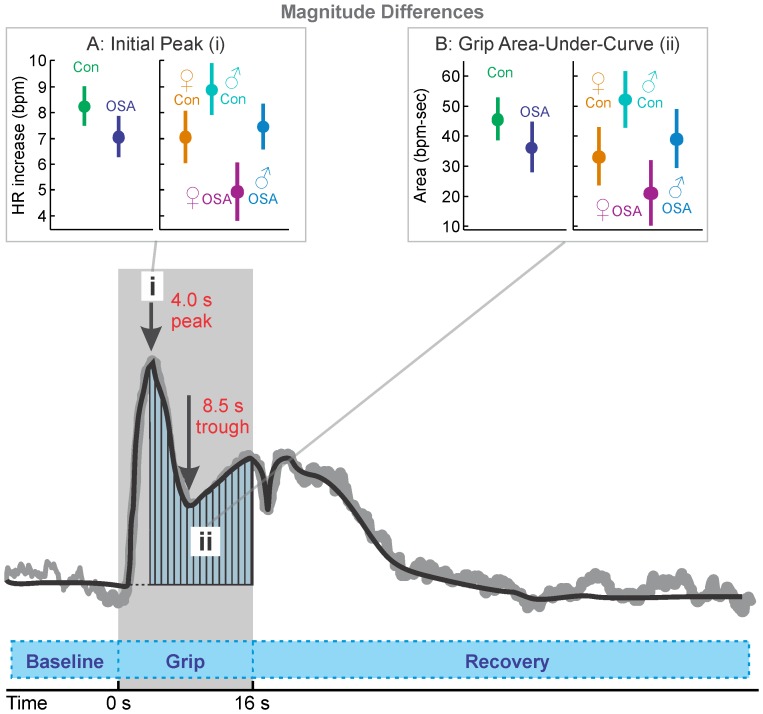
Hand grip indices. A: heart rate increase from baseline to initial peak (i); B: sustained heart rate elevation during main challenge period (area-under curve, ii). Time trend graph illustrates the mean heart rate of the 57 subjects in the control group, with a simplified representation (thick black line) overlaid on the measured values (gray line). Shaded area indicates challenge period.

These measures are intended to quantify visually apparent patterns of group differences in the mean signals, as seen in Figs. 2, 4 & 6. However, descriptive (SEM) and comparative (ANOVA) statistics are also presented for measures with individually calculated data (i.e., not delays, where were calculated from the mean signal only).

### Cold Pressor in OSA vs. Control (Mixed Female and Male)

Heart rate rose in response to the cold pressor challenge to an early peak in both groups (Fig. 1Ai, 1Aii); however, the increase in the OSA vs. control groups was muted (6.0% vs. 8.5%; Fig. 2A) and delayed (11 vs. 8 s into challenge period; Fig. 1ii). The increase was not sustained in the OSA group, but the controls showing elevated heart rates through the remainder of the challenge period (time 21–60 s, with a second peak of 7.5% increase); whereas, the OSA subjects showed only six time-points of marginally-significant increases during that period (and a maximum 3.6% increase). Group differences were especially apparent in the latter part of the challenge period, with more than 5% greater heart rate responses in control than OSA from 40 to 46 s. During recovery, the heart rate dropped in both groups to a nadir (OSA −6.0% at 33 s into the recovery, control −6.2% at 35 s into the recovery). However, the OSA group returned to baseline, starting from approximately 70 s into recovery; whereas, the control group remained below baseline levels for most of the 120 s recovery period.

### Cold Pressor in Females

Female OSA and control subjects showed an initial heart rate increase 24 s into the challenge period (Figs. 1Bi and 1Bii), with the OSA group showing a greater change (13.2% vs. 9.3%), but a delay (2.5 s; Table 3). However, while the control females showed a rebound to a sustained increase in heart rate for the remainder of the challenge, the OSA group showed a decline at or close to baseline from 32 s into the challenge, with significant group differences, largely within the last 10 s (Fig. 1Bii).

### Cold Pressor in Males

Male OSA subjects showed a first heart rate peak vs. controls that was muted (4.8% at 11 s vs. 9.6% at 7 s; Fig. 1Bii, Fig. 1Biii) and delayed (0.9 s; Table 3). After 6–14 s, the OSA group returned to baseline, while the control group showed a sustained higher level throughout the remainder of the challenge period. However, during recovery, both groups showed similar patterns of change, with a nadir of over 6% below baseline 33–35 s into the recovery, and no sustained group difference.

### Hand Grip Summary

The hand grip elicits a rise in blood pressure from activation of the hand musculature and voluntary effort. All groups showed a rapid heart rate increase at the start of the hand grip task, followed by a drop to a sustained, elevated level relative to baseline, consistent with sympathetic activation (Figs. 3i, 3ii). Release led to a gradual decline to baseline levels. The magnitude indices (Table 3 and Fig. 4A) showed a lower heart rate rise in OSA to the initial peak, with both females and males exhibiting a lower increase, relative to their control counterparts. The strain elevation (area under the curve, Fig. 4B) was similarly lower in the female OSA subjects especially. The timing delay to the first peak was apparent in the male OSA group, but not the female patients, who showed a more rapid (by 0.36 s) increase to the initial peak (Figs. 3Bi, 3Bii, 3Biii).

### Hand Grip in OSA vs. Control (Mixed Female and Male)

Assessment of the time-trends showed that the peak heart rate occurred at 5 s in both groups (Fig. 3Ai and Aii), but the magnitude of change was lower in OSA (6.7% vs. 7.6% in controls; Fig. 4A), and slightly delayed (0.2 s). The control group also showed a gradual increase in the latter half of the challenge period, with significant group differences over 13–16 s (Figs. 3Ai, 3Aii). Both groups returned to baseline 18 s into the recovery, but the control group showed higher heart rate changes during some of that recovery (i.e., at 4 and 8–12 s).

### Hand Grip in Females

Females in both groups showed an initial heart rate peak at 4 s, which in OSA patients was lower than female controls (3.3% vs. 5.8%; Fig. 3Bii, 4A), but with no delay. The female OSA heart rate returned to baseline levels for most of the second half of the challenge, in contrast with the consistent elevation in female control subjects. Both groups showed a heart rate rebound in the recovery period, but the OSA peak was delayed by 3 s compared with that of the control group (8 s vs. 5 s).

### Hand Grip in Males

Males in both groups showed an initial heart rate peak at 5 s (1 s later than the females), with the increase lower in OSA than control subjects (7.4% vs. 8.6%; Fig. 3Biii, 4A). Both groups maintained an elevated heart rate for the remainder of the challenge period, and into the recovery period (to 17 s for OSA and 21 s for control). However, the control group showed higher increases than the OSA in the last 4 s of the challenge (with the greatest group difference of 3.2% occurring in the last time-point of the task), and for most of the first 12 s of recovery.

### Valsalva Maneuver Summary

**Figure 5:**
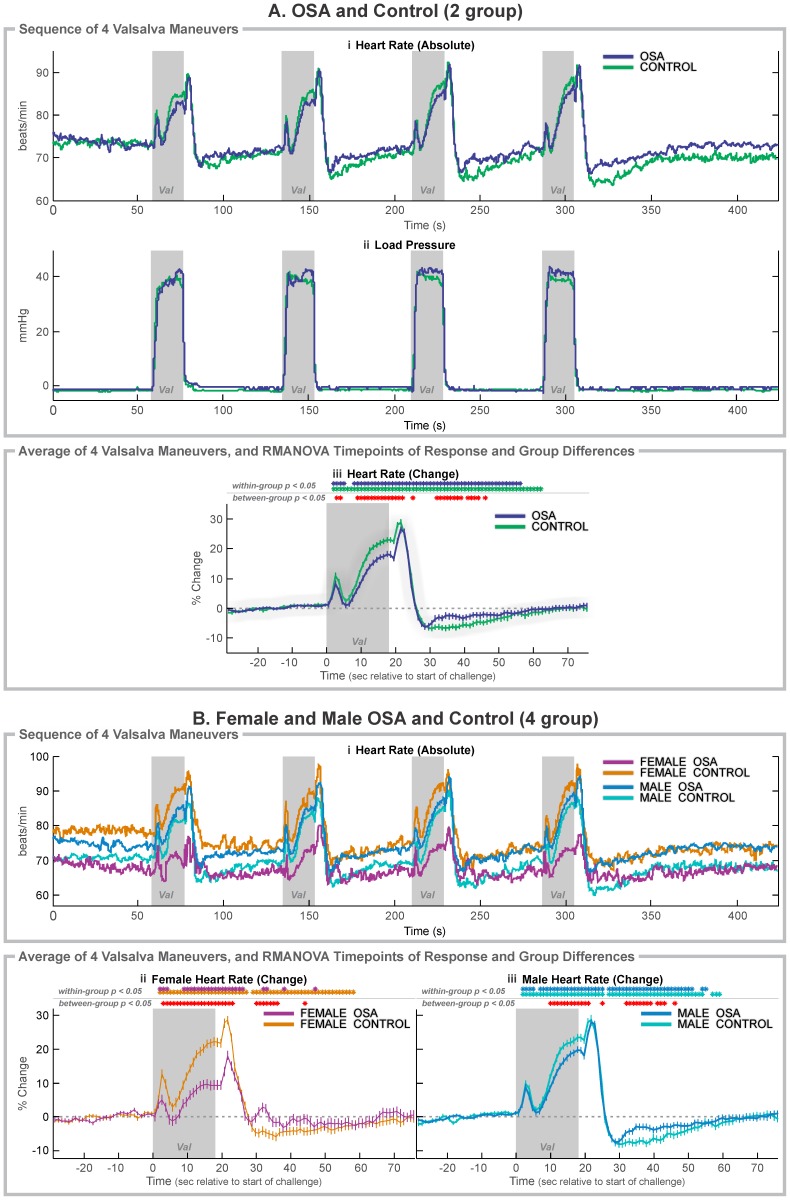
Valsalva maneuver heart rate responses. A: 37 OSA and 57 control subjects; B: 6 OSA and 20 control female subjects, and 31 OSA and 37 control male subjects. Ai, Bi, Raw heart rate and Aii load pressure during the complete sequence. Aiii,Bii, Biii: change in signals relative to baseline (group mean ± SE) averaged over 4 challenges, with time-points of significant increase or decrease relative to baseline within-group, and time-points of between-group differences (RMANOVA, *p*<0.05). Shaded areas (Val) indicate challenge periods.

**Figure 6:**
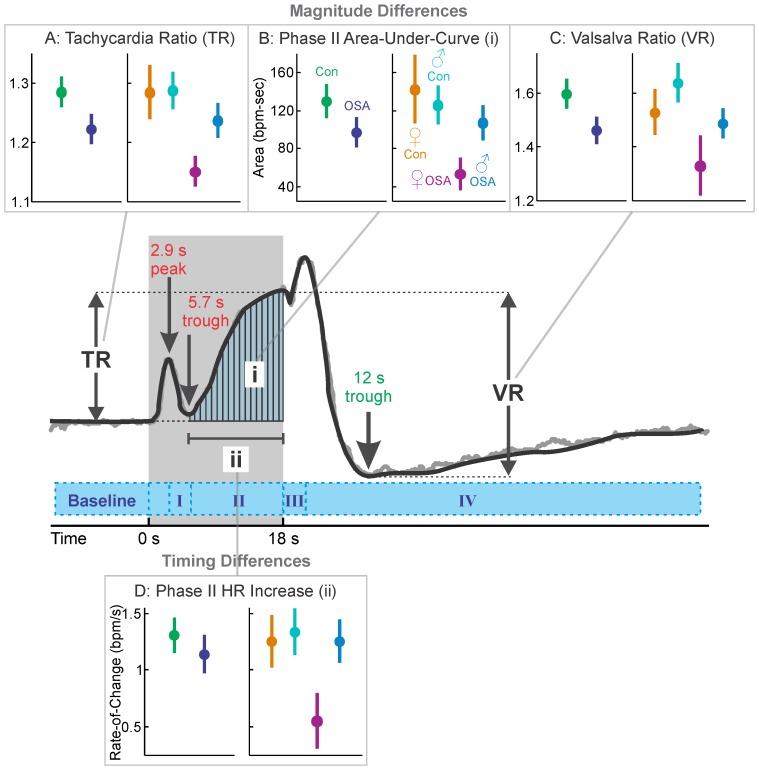
Hand grip indices. A: tachycardia ratio (TR; peak during i/baseline); B: heart rate increase from baseline to initial peak (i); C: Valsalva ratio (VR; peak during ii/minimum during recovery); D: rate of change (slope) during phase II increase (iii). Time-trend graph illustrates the mean heart rate of the 57 subjects in the control group, with a simplified representation (thick black line) overlaid on the measured values (gray line). Shaded area indicates challenge period.

The Valsalva maneuver elicits a sequence of blood pressure changes including a rapid increase in pressure, and upon air pressure release, a rapid decline, then recovery. The characteristic pattern of heart rate changes reflects adaptation to the blood pressure fluctuations, with sustained rate increases in the mid-onwards strain period (phase II), and upon release, rapid, brief increase (phase III), followed by a sustained fall below baseline, then recovery (phase IV) [43]. The Valsalva maneuver elicited similar patterns of response in all groups, with the characteristic four phases, but the extent of change was muted in the OSA patients (Fig. 5). The classical Valsalva and tachycardia ratio indices (Table 3 and Fig. 6A, 6C) illustrate the lower response in the OSA group, with the patient-control separation also apparent in the female and male groups. The phase II elevation (area under the curve; Fig. 6B) highlighted the much-lower OSA female sympathetically-drive heart rate increase, which was also associated with a greatly reduced rate-of-increase in that group. Delayed responses during the phase III rebound were in the order of 1 s.

### Valsalva Maneuver OSA vs. Control (Mixed Female and Male)

The time-trends showed that the pre-phase I heart rate peak (associated with inspiration to increase thoracic pressure prior to expiratory effort) occurred at 3 s in both groups (Fig. 5Ai and 5Aiii), but was lower in OSA vs. controls (8.2% vs. 10.8%). The trough prior to the sympathetic portion of phase II occurred at 6 s, and both groups increased heart rate for the remainder of the challenge, but with the control changing more than the OSA group (23.0% vs. 18.2%; Fig. 6B). The rebound at onset of phase III in OSA was slightly (26.3% vs. 28.7%) and delayed (1.1 s) (Fig. 5 Aiii), and the OSA group returned to baseline earlier than the control group (39 vs. 45 s into recovery).

### Valsalva in Females

All OSA and control groups showed the characteristic pattern of heart rate changes, but the OSA females showed substantially muted responses, with significant group differences for most of the challenge and 20 s into the recovery (Fig. 5Bi and 5Bii). For example, the initial pre-phase I peak at 3 s was 5.1% in female OSA vs. 12.9% in female controls, and the maximum phase II increase (time 18 s) was only 9.8% in OSA vs. 21.6% in controls. The recovery phase III peak was delayed in OSA (0.7 s; Table 3). The phase IV recovery was greatly muted in OSA, with a return to baseline within 10 s of release vs. after 40 s for the control group.

### Valsalva in Males

The male OSA and control subjects showed the typical pattern of heart rate changes, with the OSA group being slightly muted, but to a much lesser degree than their female counterparts (Figs. 5Bi, 5Biii). Group differences emerged in the latter part of phase II, with the greatest separation occurring at 13 s into the challenge period, where the OSA increase was 15.0% vs. 19.6% in controls. The recovery phase III peak was delayed in OSA (1.3 s). The group differences disappeared during the initial phase IV period (the decline in heart rate), but in the post-decline portion of phase IV, the OSA group showed a more rapid return toward baseline, with significant group differences being present at 14–28 s into the recovery.

### Additional Findings

The resting heart rate varied in the combined female and male control group, with a baseline rate before the cold pressor of approximately 70 bpm, but higher prior to the hand grip, and above 70 bpm prior to the Valsalva maneuver (Figs. 1Bi, 3Bi & 5Bi). These differences were prominent in the control females, who, relative to the other three groups, showed high resting heart rates during baseline before all challenges. Before the Valsalva maneuver, which was always performed before the hand grip and cold pressor challenges, the absolute heart rate was the same in OSA and control subjects. However, heart rate was lower in controls prior to the hand grip and cold pressor challenges.

## Discussion

Heart rate responses to autonomic challenges are disrupted in OSA patients, and the patterns of alterations differ between the sexes. The common pattern of heart rate differences in OSA was a reduction in magnitude of responses, and a delayed, or slower rate of change in those responses. A post-hoc separation by sex highlighted the differing female and male patterns in healthy control subjects, as well as the different amplitude and time-courses of OSA-control effects within sexes, with female patients showing greater impairments from their male counterparts. The consequence of failing to adequately adapt to typical day-to-day challenges (e.g., body position changes, straining, temperature change), as mimicked in part by the standard tests used in this study, would likely include acute periods of reduced tissue perfusion or oxygenation. Moreover, knowing the close integration between upper airway muscle tone and transient changes in blood pressure [44], delays in blood pressure responses have the potential to further exacerbate the impaired respiratory muscle action in OSA. Impaired dynamic autonomic nervous system responses, as reflected in blunted heart rate responses to acute challenges, is a risk factor for hypertension and cardiovascular disease, as well as other chronic illnesses associated with autonomic dysfunction [45]–[48]. The inability to modify regulation of vascular flow from postural movements can contribute to commonly-observed deficits, such as those found with orthostatic hypotension [18], [49], or the failure to alter blood pressure and heart rate according to respiratory movements (reflected as low heart rate variability, a predictor of sudden death in certain conditions [50], [51]).

All three challenges highlight the muted heart rate response and alterations in recovery to autonomic provocations in OSA. Similar patterns appear in other OSA populations to various autonomic challenges [7], [9], [10], [52], [53]. However, the similarities and differences across the three challenges suggest greater impairment of particular components of the autonomic nervous system.

The most notable similarities across the three distinct autonomic challenges are that OSA patients increase heart rate to a lesser extent that controls during sympathetic phases, whereas heart rate declines due to sympathetic withdrawal or parasympathetic activation are virtually identical to those in healthy subjects. The Valsalva maneuver showed similar principally parasympathetically-mediated heart rate declines in both OSA and controls (although with modest impairment in female OSA patients). For the same task, the heart rate changes induced as a baroreflex-mediated response to the mechanical pressure changes at challenge onset were similar between groups. However, the latter part of the forced expiration during the predominantly-sympathetic activation period elicited large group differences. The cold pressor challenge was also remarkable in the consistently lower heart rate response in the OSA groups throughout the challenge period, again reflecting the weakened sympathetic phase response. In contrast, the heart rate decline after removal of the foot from cold water was similar in all groups. The pattern of heart rate changes to the hand grip also showed lower increases in OSA groups later in the challenge during the more-sustained sympathetic period, together with a delayed initial increase. A greater importance of sympathetic vs. parasympathetic dysfunction in OSA has been previously proposed [19]. In the present study, the similarities across challenges of heart rate declines overlapping those in controls, and of muted rate increases during the sympathetic phases, suggest preferentially sympathetic over parasympathetic impairments in OSA.

The most notable differences between challenges in the patterns of OSA impairments were in timing characteristics. An initial heart rate increase occurred in all three challenges, but the heart rate rise was delayed in the cold pressor and hand grip tasks, but not in the Valsalva maneuver. Thus, integration of the voluntary regulatory component, or some aspect of afferent activity in the peripheral limb action in the hand grip task, and temperature afferent input in the cold pressor challenge may be delayed in OSA. Since the Valsalva maneuver also involves a voluntary effort (and timing is little changed), the impaired temporal processing in the hand grip challenge suggests afferent processes, rather than voluntary motor areas may be more affected. The cold challenge recruits medullary nuclei for wetness, cold, and pain stimuli, and projects to intermediary periaqueductal gray and other intermediary structures. Since structural alterations in pathways to those structures are prominent in OSA [6], [11], inadequate function of those brain areas may lead to slower processing and contribute to the observed slower responses to the pain and cold challenges.

Brain alterations leading to impaired autonomic regulation may interfere with responses to more than just pain and cold stimuli. The number of studies showing central alterations and disrupted brain function in OSA patients within structures that regulate autonomic actions is now substantial [4]–[11], [54]–[62]. Those changes may underlie the impaired neural control of vascular responses. Neuronal sites and connecting white matter are damaged in OSA [5], [6], [11], with some of the changes likely present also in glial cells [63], [64]. Impacted regions that play critical regulatory roles in autonomic functions include the anterior cingulate and ventral medial prefrontal cortices, the hippocampus, the left and right insular cortices and connecting fiber tracts, and the cerebellar cortex and deep nuclei. Lesion, stimulation, stroke and fMRI studies in humans and animals demonstrate that all these regions influence outflow from medullary and hypothalamic regions [65]–[73]. We previously demonstrated that the challenges used in the present study elicit responses within the hippocampus, cingulate and insular cortices, with regional differences between subregions of the insula [20], [74]. The majority of the brain areas affected in OSA have excitatory and inhibitory interactions with each other in modulating autonomic outflow; thus, pathology in any of multiple autonomic sites has the potential to modify sympathetic and parasympathetic action. A final point is that the brain alterations may be reversible [75], with some evidence that the structural changes seen on MRI scans reflect an acute inflammatory state [11], potentially reversible with CPAP treatment [56]. Recovery of affected brain autonomic regulatory regions with CPAP is consistent with findings of autonomic status being partially restored with treatment [76]–[78].

Heart rate responses in OSA were delayed, in addition to being muted. The initial peak response to the cold pressor challenge was 3 s later in the patient group (38% time-delay), and perhaps derived from damage to the medullary integrative circuitry for pain and arousal systems accompanying pain. Timing differences did not appear to the hand grip, but the Valsalva patterns highlighted a delayed peak during the compensatory heart rate increase upon release (1 s, or 33% time-delay), as well as a later plateau effect (at least in males) during the sympathetic period. These findings suggest impaired central timing coordination, possibly resulting from cerebellar injury found in OSA [5], [6], [8], [54], [79], since that brain region serves important coordination roles for autonomic regulation [50], [80], [81], and showed delayed fMRI signals to a Valsalva challenge in an earlier study [10], in addition to its well-known motor coordination functions.

Female OSA patients showed more substantial deviations from their healthy counterparts than OSA males, although the generalizability of these findings is limited by the small numbers in some groups. The Valsalva maneuver elicited markedly reduced heart rate responses in the female OSA group from the initial pressure-induced heart rate peak. This diminished response was all the more remarkable in that females with OSA had low baseline heart rate, and so were less likely to suffer from a ceiling effect that could limit the extent of increase. No subjects reported using any cardiovascular or other autonomic medications; thus, these findings can be assumed to reflect intrinsic physiology. All subjects performed the task with similar expiratory pressures, so challenge variation should not have contributed to the response differences. Also noteworthy were the highly altered heart rate responses to the cold pressor challenge in female OSA patients; while female patients initially showed a large increase similar to the control group, the male patients experienced a muted, delayed initial increase. Only female OSA patients did not sustain the heart rate increases to the hand grip challenge. In contrast, the heart rate declines during recovery periods for all three challenges were similar to the control group patterns for OSA female and male subjects. The pattern that emerges is one of substantially altered regulation in the female OSA patients, with at times different patterns (as opposed to just differing magnitudes and timing) of response from the healthy control subjects. In contrast, the male OSA patients showed a predominantly muted, and sometimes delayed version of the healthy response. The sex differences in heart rate responses may arise from greater, or differently-sited neural impairments in the female OSA patients [26]. The present sample was a subset of the female OSA patients showing structural brain alterations in the earlier study [26].

Resting heart rate during baseline periods prior to initiation of the challenges tended to be higher in OSA patients than control subjects (for hand grip and cold pressor, but not Valsalva). High resting heart rate in many conditions is associated with high sympathetic tone, which is present in OSA [18]. However, this pattern was not consistent between the sexes, with the female control subjects showing the highest resting baseline heart rates, but the female OSA patients showing similar levels to male controls. Thus, the interpretation of the resting heart rate differences is unclear; it may be the case that a sex difference exists with anxiety associated with MRI scanning (anxiety symptoms are higher in female than male OSA patients [82]).

### Considerations

OSA is a heterogeneous condition, with several confounding factors known to influence central nervous system and autonomic functions, which include obesity, but also depression [83] and anxiety [84]. We could not perform a comprehensive assessment of influences of BMI and other variables in this paper, but we do note that the distributions of control and OSA values overlapped substantially, in spite of a significant mean difference. Thus, at least some of the reported differences are likely due to OSA, even if other factors also contribute.

The female OSA group had only six subjects, and typically showed high variability. An additional four female OSA subjects were excluded due to failure to successfully perform challenges, and data artifact. The small number of female subjects limits the generalizability of the findings, and the sensitivity of the statistical analysis. The higher proportion of excluded subjects raises the possibility that the tasks are more challenging for female OSA subjects, either due to limitations in strength or, as experienced by a few subjects, difficulty in sealing lips around the tube.

Migraine was not an exclusion criterion, even though that condition is associated with altered autonomic function. However, no subjects were taking autonomic-active medications, including pain-relieving or preventative migraine prescriptions.

The hand grip challenge involved a non-quantitative magnitude of grip force, and was a subjective, non-isometric challenge. Ideally, the protocol would involve sustaining a grip pressure at a percentage of maximum. Thus, unlike the Valsalva and cold pressor challenges, the hand grip effort should not be considered a physically equivalent challenge across subjects or groups.

### Conclusions

Heart rate responses are blunted and delayed in OSA patients; those differences are exacerbated in females. The blunted heart rate changes in OSA were observed in all challenges, and were principally a failure to increase heart rate to the same extent as healthy control subjects during sympathetic-dominant periods, whereas heart rate declines during recovery were of similar magnitudes in patient and control groups. The delayed heart rate responses occurred in response to challenges requiring integration of cold temperature and pain input and limb proprioceptive input, suggesting medullary cold and wetness processing or arousal integration projections were affected for the cold pressor challenge and complex proprioceptive processing in thalamic and other afferent sites for the hand grip task. Such functional impairments are consistent with the known structural brain changes in OSA. The exacerbation of impairments in female vs. male OSA may reflect the additional brain alterations found in female patients. The overall finding is one of reduced, and sometimes delayed, principally sympathetically-mediated heart rate responses to autonomic challenges in OSA. The autonomic deficits in OSA likely reduce the effectiveness of regulation of blood pressure changes and movement, as well as brain perfusion. While both brain structural changes and some sympathetic abnormalities are partially normalized with CPAP, the effect of treatment on acute autonomic responses is unclear.
